# Efficacy of Metastasis-Directed Radiation Therapy to Prolong Systemic Therapy for Patients with Oligoprogressive Metastatic Breast Cancer

**DOI:** 10.3390/cancers17132153

**Published:** 2025-06-26

**Authors:** Alexis LeVee, Hannah Young, Stephanie Yoon, Scott Glaser, Shengyang Wu, Joanne Mortimer, Jose G. Bazan

**Affiliations:** 1 Department of Medical Oncology, City of Hope National Medical Center, Duarte, CA 91010, USA; alevee@coh.org (A.L.);; 2 Department of Radiation Oncology, City of Hope National Medical Center, Duarte, CA 91010, USA

**Keywords:** oligoprogressive, oligometastases, radiation therapy, metastasis-directed therapy, breast cancer

## Abstract

Background: Clinical trials have shown mixed results regarding the benefit of metastasis-directed radiation therapy (MDRT) in oligoprogressive (OP) metastatic breast cancer (MBC), leading to ongoing debate about its role. This study aimed to investigate whether MDRT can prolong the duration of systemic therapy for ≥6 months in patients with OP MBC. Methods: This retrospective cohort study included patients with MBC who received MDRT for OP disease between December 2017 and March 2023. Patients who received MDRT to the brain were excluded. Medical records were reviewed through July 2024. The primary endpoint was the proportion of patients remaining on the same systemic therapy for ≥6 months post-MDRT. Results: In total, 52 patients with OP MBC treated with MDRT were included, with 36 (69%) with HR+/HER2- disease, 10 (19%) with HER2+ disease, and 6 (12%) with TNBC. Among the 47 patients with follow-up data available, 28 (60%) remained on their systemic therapy at 6 months, including 65% (22/34) of patients with HR+/HER2- disease, 56% (5/9) with HER2+ disease, and 25% (1/4) with TNBC (*p* = 0.30). Among the 38 patients with a follow-up time of at least 1 year post-MDRT, 47% (18/38) remained on the same systemic therapy. The median time to next systemic therapy and median PFS were 6.9 months (95% CI, 5.7–14.7) and 6.2 months (95% CI, 4.1–9.7), respectively. Conclusions: Over half of patients with OP MBC remained on the same systemic therapy for at least 6 months following MDRT, which suggests that MDRT may help prolong systemic therapy duration for select patients.

## Simple Summary

Clinical trials have shown mixed results regarding the benefit of metastasis-directed radiation therapy (MDRT) in oligoprogressive (OP) metastatic breast cancer (MBC), leading to ongoing debate about its role. This retrospective cohort study aimed to investigate whether MDRT can prolong the duration of systemic therapy for at least 6 months in patients with OP MBC. We found that 60% of patients with OP MBC remained on the same systemic therapy for at least 6 months following MDRT, which suggests that MDRT may help prolong the duration of systemic therapy for select patients.

## 1. Introduction

Radiation therapy (RT) in patients with metastatic breast cancer (MBC) is primarily used for palliative purposes, but emerging data suggests that it may have a role in improving systemic disease outcomes for select patients. In patients with oligometastatic (OM) disease, defined as the presence of a limited number of metastatic lesions, several large clinical trials have reported that metastasis-directed RT (MDRT) can prolong progression-free survival (PFS) and overall survival (OS) in several types of cancer including prostate cancer and non-small cell lung cancer (NSCLC) [1,2,3,4,5]. The benefit of MDRT in patients with OM breast cancer is less clear, with two randomized prospective studies showing no benefit to MDRT [6,7].

RT is also being investigated in patients with oligoprogressive (OP) MBC, defined as progression and/or new metastases in a few lesions (generally, 1–5) while the rest of the disease remains stable. OP in MBC is relatively common, occurring in approximately 21–30% of patients [8,9]. The rationale for this approach is that OP occurs as a result of tumor heterogeneity, in which treatment-resistant clones drive progression of a few lesions [10]. Local RT to these areas may eradicate these treatment-resistant clones, potentially allowing patients to remain on their otherwise effective systemic therapy for a longer duration.

Studies evaluating MDRT for OP in breast cancer are limited [11,12,13,14,15,16,17,18]. While several phase II clinical trials in NSCLC showed improvements in PFS and OS with MDRT for OP disease [17,19,20], studies of MDRT for OP MBC are inconclusive. The randomized phase II STOP trial which included patients with a variety of solid tumors identified no difference in PFS or OS in patients treated with SBRT compared to the standard-of-care [16]. However, in the subgroup analysis, patients with breast cancer did have a significant improvement in PFS with SBRT, although the number of patients with breast cancer included in the study was small (n = 12) [16]. The randomized phase II CURB study which enrolled 47 patients with OP MBC of all subtypes to stereotactic body radiotherapy (SBRT) compared to the standard-of-care did not find an improvement in PFS [17].

In contrast, the single-arm phase II AVATAR trial that included only patients with hormone receptor-positive (HR+)/human epidermal growth factor receptor 2-negative (HER2-) advanced breast cancer demonstrated promising results [18]. SBRT to OP lesions led to 47% of patients remaining event free, as defined by a time to change in systemic therapy after SBRT or any progression within 6 months or in >3 lesions, for at least six months [18]. This suggests that MDRT may be an effective treatment strategy for certain patients with OP MBC, particularly those with HR+/HER2- breast cancer.

Given the lack of clarity with regard to the role of MDRT in OP MBC, we conducted a retrospective, single institution cohort study to determine clinical outcomes of MDRT in OP MBC.

## 2. Methods

We retrospectively identified patients with MBC at City of Hope Comprehensive Cancer Center through the electronic medical record who received extracranial MDRT for OP to 1–4 lesions with a primary goal of local control of the targeted lesions. The upper limit of 4 for number of OP lesions was chosen due to prospective data from NRG BR001 that demonstrated the safety of delivering MDRT to up to 4 metastatic lesions at one time [5]. Patients were excluded from the study if they received MDRT to the brain or radiation for palliative intent for pain control. Among patients that received extracranial MDRT to 1–4 lesions, records were reviewed through manual chart review to confirm OP, which was defined as progression and/or new metastases in 1–4 lesions as identified on imaging scans. A goal of MDRT to the OP lesion was confirmed through the medical record. All patients received photon-based radiation with intensity modulated radiation therapy technique. The primary endpoint was the proportion of patients that remained on their systemic therapy from time of MDRT for ≥ 6 months. Secondary endpoints included the proportion of patients that remained on the same systemic therapy from time of MDRT for ≥12 months, time to next line of systemic therapy, time to local progression, PFS, and OS. Time to local progression was defined as time to progression of the treated lesion(s). PFS was defined as time from systemic disease progression or death from any cause. Medical records were reviewed through July 2024, at which point data abstraction was cut off. Patients were censored at the date of last follow-up.

Demographic information, tumor characteristics, and treatment data were collected from the electronic medical record. For patients with multiple episodes of MDRT for OP, data from the time of first MDRT for OP was collected. Toxicity was evaluated using the Common Terminology Criteria for Adverse Events (CTCAE) version 5.0. The biologically effective dose (BED) was calculated using the linear-quadratic model with both an α/β = 3 Gy and α/β = 10 Gy [21]. PFS and OS were calculated from the time of MDRT.

Univariate logistic regression was performed to identify factors associated with maintenance of systemic therapy at 6 months. Cox proportional hazards regression was performed for time to next line of systemic therapy, time to local progression, PFS, and OS. Survival probability distribution was calculated using the Kaplan–Meier method. A threshold of 0.05 was used to assess significance in all statistical analyses. All analyses were conducted in SAS9.4. This study was approved by City of Hope Comprehensive Cancer Center Institutional Review Board and was exempt from participant consent.

## 3. Results

Between December 2017 to March 2023, 52 patients met the inclusion criteria and their characteristics are summarized in Table 1. All patients were female, with a median age of 62.1 years (interquartile range [IQR], 51.4–66.9) [Table 1]. The majority (69%) of patients had HR+/HER2- disease, followed by HER2+ disease (19%), and triple-negative breast cancer (TNBC, 12%).

As part of our inclusion criteria, all patients had to receive at least 1 line of prior systemic therapy and subsequently experience OP. At the time of initial diagnosis, most patients had early-stage disease, while 27% had de novo metastatic disease (Table 1). In patients with recurrent disease, the median time from initial diagnosis to metastatic disease was 50.8 months (IQR, 27.9–101.2). The median time from metastatic disease to OP was 32.8 months (IQR, 11.5–52.5). Prior to MDRT, 60% of patients received only 1 line of systemic therapy.

At the time of MDRT, over half (58%) of patients had oligometastatic disease, with ≤4 metastatic lesions. The most common location of metastases at the time of MDRT was bone (67%), lung (37%), lymph nodes (37%), liver (17%), and previously treated or stable brain metastases (12%). At the time of MDRT, 48% of patients were on a cyclin-dependent kinase (CDK) 4/6 inhibitor, followed by HER2-targeted therapy (17%), chemotherapy (17%), and other (14%).

Table 2 describes the radiation therapy treatment data to the OP lesions. MDRT was delivered to 1 lesion in 40 (77%) patients, 2 lesions in 9 (17%) patients, and 3 lesions in 3 (6%) patients. The most common site of OP treated with MDRT was bone (58%), followed by lung (14%), lymph node (12%), liver (10%), and breast (8%). The median prescribed total dose was 30 Gy (IQR, 27–45) delivered in median fraction size 8 Gy (IQR, 5–10). The median BED delivered (α/β = 3) was 108 Gy (IQR, 7100–14,700) and the median BED (α/β = 10) was 52 Gy (IQR, 41–73.5). No patients experienced acute or late grade 3 or higher toxicity related to MDRT.

Follow-up data for disease outcomes was available in 47 of 52 patients with a median follow-up time of 19.5 months (IQR, 13.9–28.6) from the end of MDRT. Of the 47 patients with follow-up data, 37 (79%) patients experienced distant progression, 4 (9%) experienced local progression, and 10 (21%) remained event-free with neither distant or local progression at time of follow-up. Overall, 60% (28/47) of patients remained on their systemic therapy at 6 months, of which 65% (22/34) had HR+/HER2- disease, 56% (5/9) HER2+ disease, and 25% (1/4) TNBC (*p* = 0.3). Of note, only 4 patients with TNBC had sufficient follow-up data for disease outcomes following MDRT, which limited subgroup analyses in this population.

Among the 43 patients with a follow-up time of at least 6 months post-MDRT, 65% (28/43) of patients remained on their systemic therapy at 6 months, of which 69% (22/32) had HR+/HER2- disease, 63% (5/8) HER2+ disease, and 33% (1/3) TNBC (*p* = 0.5). The rates of continuation of systemic therapy for ≥6 months were similar across patients who had previously received 1 line of systemic therapy (59%, 16/27), 2 lines of systemic therapy (50%, 4/8), or >2 lines of systemic therapy (67%, 8/12) [*p* = 0.8].

**Figure 1:**
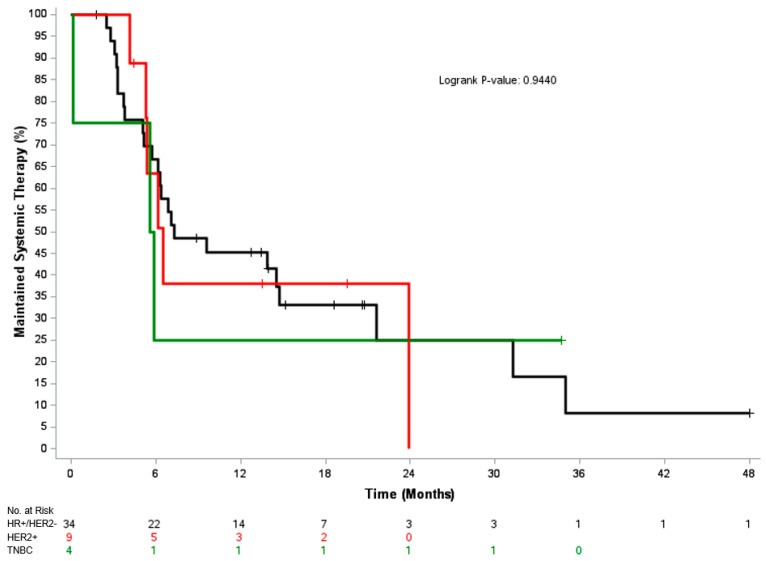
Kaplan–Meier curve for time to next systemic treatment according to breast cancer subtype. Abbreviations: HR, hormone receptor; HER2, human epidermal growth factor receptor 2; TNBC, triple-negative breast cancer.

**Figure 2:**
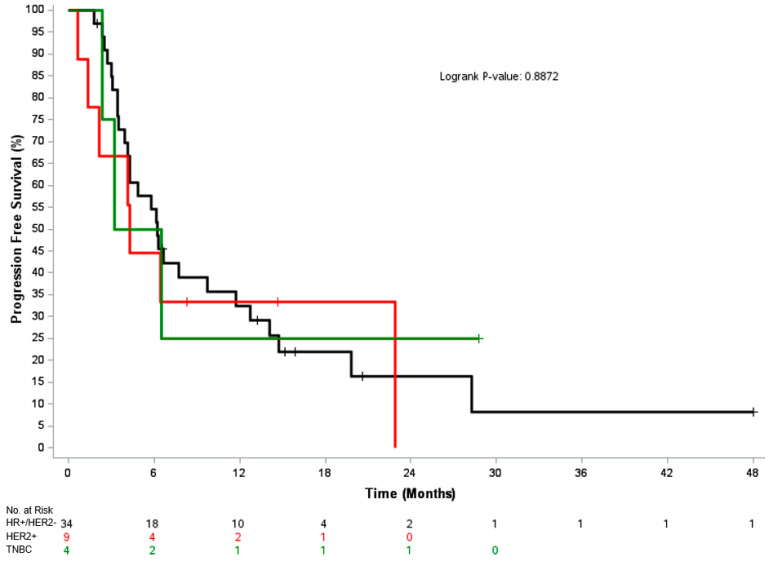
Kaplan–Meier curve for progression-free survival according to breast cancer subtype. Abbreviations: HR, hormone receptor; HER2, human epidermal growth factor receptor 2; TNBC, triple-negative breast cancer.

**Figure 3:**
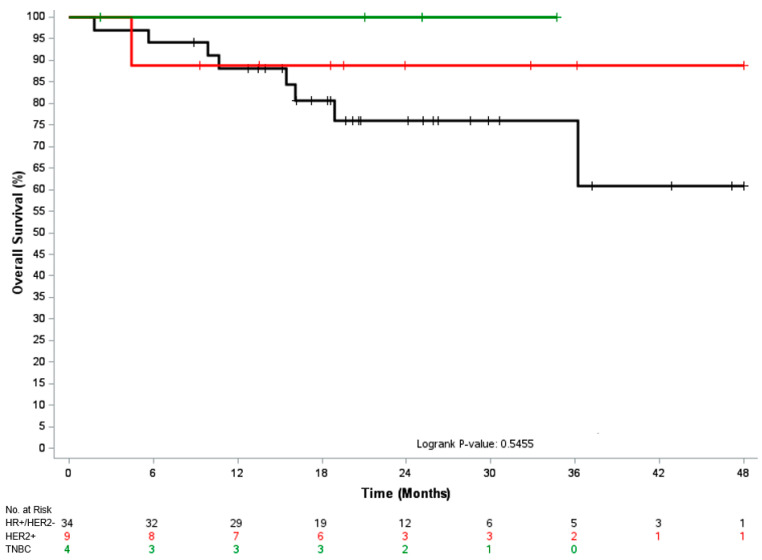
Kaplan–Meier curve for overall survival according to breast cancer subtype. Abbreviations: HR, hormone receptor; HER2, human epidermal growth factor receptor 2; TNBC, triple-negative breast cancer.

The median time to change in systemic therapy and median PFS were 6.9 months (95% CI, 5.7–14.7) and 6.2 months (95% CI, 4.1–9.7), respectively. Median OS was not reached, and the 1-year OS post-MDRT was 89%. In the 38 patients that had reached a follow-up of at least 1 year post-RT with known disease status, 47% (18/38) remained on their systemic therapy from time of MDRT, including 50% of HR+/HER2- patients (14/28), 43% of HER2+ patients (3/7) and 33% of TNBC patients (1/3) [*p* = 0.8]. Figure 1, Figure 2 and Figure 3 depicts the time to next systemic treatment, PFS, and OS according to breast cancer subtype.

Univariate logistic regression analysis for maintenance of systemic therapy for at least 6 months and cox proportional hazards regression for time to next line of systemic therapy is summarized in Table 3. Age, OM at time of RT, de novo metastatic at diagnosis, number of prior treatment lines, breast cancer subtype, bone-only disease, number of OP lesions treated, and BED3 and BED10 were not associated with maintenance of systemic therapy for at least 6 months and time to next line of systemic therapy.

Table 4 demonstrates Cox proportional hazards regression for local control, PFS, and OS. A higher BED3 (hazard ratio [HR], 0.99; 95% confidence interval [CI], 0.99–1.00; *p* = 0.02) and higher BED10 (HR, 0.99; 95% CI, 0.97–1.00; *p* = 0.04) were associated with improved PFS. A higher BED3 was associated with improved local control (HR, 0.99; 95% CI, 0.99–1.00; *p* = 0.02). No other clinical variables were associated with improved local control, PFS, or OS.

## 4. Discussion

This study contributes to the growing body of evidence that MDRT may provide an opportunity for select patients with OP MBC to continue effective systemic treatment while targeting treatment-resistant disease. In this study, more than half (60%) of patients remained on their systemic therapy for at least 6 months following MDRT. Patients with HR+/HER2- and HER2+ disease were more likely to remain on their systemic therapy at 6 months following MDRT, with 65% and 56% of patients, respectively, remaining on their systemic therapy at 6 months post-MDRT, compared to 25% of those with TNBC. However, given the lack of a control group to compare the benefit of MDRT to next line of systemic therapy without MDRT, these findings may be the result of inherit differences in prognosis of the different subgroups. In addition, due to the small sample size which may have limited the statistical significance by breast cancer subtype, particularly with respect to number of patients with TNBC, the benefit of MDRT by breast cancer subtype remains exploratory. Further randomized clinical trials are necessary to confirm the benefit of MDRT in OP MBC.

Our findings support the results of the phase II AVATAR trial, which included 32 patients with OP ER+/HER2- MBC who received first- or second-line systemic treatment with a CDK4/6 inhibitor plus an aromatase inhibitor (AI) and showed benefit of MDRT in HR+ MBC [18]. The AVATAR trial demonstrated that 47% of patients remained event-free at 6 months, with a median PFS of 10.4 months [18]. In addition, 46% of patients remained unchanged on systemic therapy at 12 months [18]. In contrast, the phase II CURB trial, which evaluated the addition of MDRT to systemic therapy and included patients with OP MBC of all subtypes (n = 47; 44%) and NSCLC (n = 59; 56%), found improved PFS for patients with NSCLC treated with MDRT, but not for patients with MBC [17]. Similarly, the phase II STOP trial which included 90 patients with all solid tumor histologies (13% with breast cancer, n = 12) showed no difference in PFS or OS in patients treated with MDRT versus the standard-of-care [16]. However, on subgroup analysis, patients with breast cancer demonstrated a significant improvement in PFS with MDRT [16].

The difference in outcomes across these phase II trials can largely be attributed to differences in patient populations, particularly with respect to breast cancer subtype and number of prior lines of therapy [22]. The AVATAR trial included only patients with HR+/HER2- breast cancer who had received one or two prior lines of therapy and were still considered to have estrogen-sensitive disease [18]. On the other hand, the CURB study included patients with all breast cancer subtypes, with 34% with TNBC [17]. In addition, of those who received MDRT, 46% had more than five metastatic disease sites and the median lines of systemic therapies received was 4 in the standard-of-care arm and 3 in the MDRT arm [17]. As a result, the CURB study likely comprised patients with more advanced disease than the current study with more aggressive and heterogenous tumor biology, which may have resulted in higher rates of treatment resistance. Indeed, the 6-month PFS of MBC patients in the standard-of-care arm was small at 4.2 months, reflecting a set of patients with advanced disease [17]. Similarly, the STOP trial included patients with all breast cancer subtypes; however, given only 12 patients with MBC were included, it is difficult to ascertain differences in patient populations within this cohort [16].

The conflicting results among these trials may also be attributed to differences in study endpoints [22]. The primary endpoint of the single-arm AVATAR trial was event-free survival (EFS) at 6 months, as defined by a time to change in systemic therapy after SBRT, any progression within 6 months or in >3 lesions [18]. The study hypothesized that SBRT would delay a change of systemic therapy for at least 6 months for at least 25% of patients. The null hypothesis of the study was rejected and the study considered positive as 47% of patients remained event-free and on a CDK4/6 inhibitor and an AI at 6 months.

On the other hand, the primary endpoint of the CURB and STOP trials, which were both randomized studies, was median PFS, which showed no significant differences with the addition of SBRT to standard-of-care systemic therapy. However, both EFS and PFS are surrogate markers for overall survival and do not necessarily translate into clinical benefit for patients [23]. In our study, maintenance of systemic therapy as the primary endpoint may also introduce bias, influenced by subjective clinical judgements, such as minimal signs or symptoms of progression, or patient-specific factors, including preference to continue treatment or irregular routine follow-up. A change in systemic therapy due to patient-reported outcomes, as opposed to signs of progression on routine imaging, may result in improved long-term outcomes for patients [24]. These differences in endpoints are important to consider in determining the true clinical benefit of MDRT.

Given that MDRT could provide an opportunity for patients to remain on their otherwise effective systemic therapy for longer duration, it could allow patients to delay the transition to more toxic and expensive systemic therapies. As such, studies have shown that MDRT in OP disease can be a cost-effective approach for the health care system [25]. By targeting only progressing lesions, MDRT could delay or prevent the need for newer, more expensive therapies, as well as therapies that are associated with more toxicity, such as chemotherapy. It may also potentially induce a systemic therapy-free interval. Furthermore, by improving clinical outcomes and preserving quality of life, MDRT can reduce the overall burden on the health care system.

The largest study to date of MDRT in OP MBC is a prospective-retrospective, single-center cohort study of 129 patients that reported similar outcomes to the present study [26]. 77% of patients had HR+/HER2- disease, and the median lines of prior therapy was 2. The study demonstrated that patients with OP MBC derived clinical benefit from MDRT, with a median PFS of 11.3 months (95% CI, 9.1–13.5) and median time to next systemic therapy of 13.6 months (95% CI, 11.5–15.2). Other retrospective studies also suggest a similar benefit of MDRT in patients with OP MBC, particularly in patients with a solitary metastasis and those with HR+/HER2- disease [13,14,15,27].

Similarly, patients with small volume disease and HR+/HER2- disease appear to benefit the most with MDRT for OM breast cancer. In a systematic review which included 10 studies comprising 467 patients with OM breast cancer treated with MDRT, MDRT was associated with high rates of local control, with bone-only metastases significantly associated with prolonged OS [28]. In another review of 500 patients with OM breast cancer treated with RT, clinical factors predictive of a favorable response to RT included HR+/HER2- subtype, solitary metastasis, bone-only metastasis, and long metastasis-free interval [29]. These findings suggest that patients with small volume disease and HR+/HER2- subtype may benefit the most from MDRT for OM breast cancer. However, the phase II NRG-BR002 and EXTEND trials, which investigated the addition of metastasis-directed therapy to the standard-of-care in OM breast cancer, found no improvements in PFS or OS [6,7]. Notably, TNBC comprised only 8% and 18.2% of patients included in these trials, respectively, and subgroup analyses by receptor status were not reported [6,7].

In this study, no patients experienced grade 3 or higher toxicity related to MDRT. Other studies of MDRT for OP breast cancer also reported minimal toxicity from RT [13,14,18,26]. In the AVATAR trial, over half of patients experienced no treatment-related toxicity, and no grade 3 or higher toxicities were observed [18]. This confirms that MDRT in the setting of OP MBC is relatively safe and well-tolerated.

Limitations of this study include being retrospective in nature, which lacks a control group to compare the benefit of MDRT to next line of systemic therapy. It also did not allow for standardized tumor evaluations, which supported the use of maintenance of systemic therapy as our primary endpoint rather than PFS. In addition, the small sample size may have limited the power of our study to detect significance in differences in clinical outcomes according to breast cancer subgroups and to identify predictors associated with improved outcomes on univariate analysis. Because of this small sample size and the limited statistical significance on univariate analysis, multivariate analyses were not performed, which would have allowed us to account for potential confounding variables. The short follow-up time also prevented the ability to assess long-term OS benefits following MDRT. Additionally, the inclusion of different systemic therapies combined with MDRT limited our ability to understand potential synergistic effects of certain therapies with MDRT. Lastly, patients included in our study may have also been subject to selection bias, potentially representing a cohort with less advanced disease given the high rate of patients with limited metastatic sites at the time of OP and the few prior lines of systemic therapy. Nevertheless, patient selection is essential in determining which patients are most likely to benefit from MDRT, and this aligns with the current body of evidence which suggests that this subset of patients benefit more from MDRT.

Further prospective clinical trials are needed to identify the specific patient populations who benefit most from MDRT in OP MBC. Research to identify biomarkers predictive of radiation response and to recognize tumors likely to experience early distant progression are ongoing to assist in patient selection. PET radiotracers may also provide additional insight into active metastatic lesions in OP disease. For example, FES radiotracers which utilize a radiolabeled form of estradiol can detect ER+ active lesions, which can help guide MDRT in ER+ OP MBC–the subtype of breast cancer which appears to benefit from MDRT as demonstrated in the AVATAR trial. An ongoing trial is underway at our institution to explore the use of FES PET/CT imaging in combination with MDRT to improve the identification of OP in patients with ER+ MBC (NCT06260033). Additionally, several ongoing clinical trials including EXTEND-OP (NCT06367972) and COSMO (NCT05301881) are investigating RT for OP MBC. Until these prospective trials are completed, this study shows that MDRT may provide a clinically meaningful benefit to patients with OP MBC by extending the duration of systemic therapy with minimal toxicity.

## 5. Conclusions

In summary, MDRT may prolong the duration of systemic therapy for select patients with OP MBC, with over half of patients remaining on the same line of systemic therapy for at least six months. Our data adds to the growing body of literature suggesting that appropriate patient selection is crucial in identifying patients with OP MBC that may benefit from MDRT. Notably, patients with HR+/HER2- and HER2+ breast cancer subtypes were more likely to remain on their systemic therapy following MDRT, although whether this is due to inherit differences in prognosis or the effect of MDRT remains uncertain due to the lack of a control group. The benefit of MDRT by breast cancer subtype, however, was not statistically significant, potentially limited by our small sample size. Ongoing prospective studies will help clarify which patients with OP MBC will benefit from RT, and future prospective studies should focus on a single subtype of breast cancer. This data provides the basis for an institutional single-arm prospective study of MDRT in patients with ER+ MBC with the use of FES PET/CT to help confirm OP at study entry (NCT06260033).

## Figures and Tables

**Table 1:** Demographic information and clinical characteristics at time of MDRT.

	Patients (n = 52)
Female, n (%)	52 (100%)
Age at time of MDRT, median years (IQR)	62.1 (51.4–66.9)
Histology	
Ductal	44 (85%)
Lobular	4 (8.0%)
Other	3 (6%)
Unknown	1 (2%)
Stage at initial diagnosis	
1	8 (15%)
2	9 (17%)
3	14 (27%)
4	14 (27%)
Unknown	6 (12%)
Breast cancer subtype at time of OP	
HR+/HER2-	36 (69%)
HER2+ (any ER/PR)	10 (19%)
TNBC	6 (12%)
Time from initial diagnosis to metastatic disease, median months (IQR)	50.8 (27.9–101.2)
Time from initial metastatic disease diagnosis to OP, median months (IQR)	32.8 (11.5–52.5)
No. lesions at time of metastatic disease diagnosis	
≤4	31 (60%)
>4	21 (40%)
No. lesions at time of MDRT	
≤4	30 (58%)
>4	22 (42%)
Location of metastases at time of MDRT	
Bone	35 (67%)
Lung	19 (37%)
Lymph nodes	19 (37%)
Liver	9 (17%)
Brain	6 (12%)
Other (adrenal, abdomen, mediastinum, peritoneum)	5 (10%)
No. of prior lines of systemic therapy	
1	31 (60%)
2–3	16 (31%)
4–5	5 (9%)
Systemic therapy	
CDK4/6 inhibitor	25 (48%)
HER2-targeted therapy	9 (17%)
Chemotherapy	9 (17%)
PD-1/PD-L1 inhibitor	2 (4%)
Other	7 (14%)

Abbreviations: MDRT, metastasis-directed radiation therapy; OP, oligoprogression; HR, hormone receptor; HER2, human epidermal growth factor receptor 2; ER, estrogen receptor; PR, progesterone receptor; TNBC, triple-negative breast cancer; CDK, cyclin-dependent kinase.

**Table 2:** Radiation therapy treatment data.

	Patients (n = 52)
No. of OP lesions at time of MDRT	
1	40 (77%)
2	9 (17%)
3	3 (6%)
MDRT site	
Bone	30 (58%)
Lung	7 (14%)
Lymph node	6 (12%)
Liver	5 (10%)
Breast	4 (8%)
Other (Abdominal wall, adrenal gland, mediastinum)	3 (6%)
Prescribed total dose (Gy), median (IQR)	30 (27–45)
Fraction size (Gy), median (IQR)	8 (5–10)
BED a/b = 3 (Gy), median (IQR)	108 (71–147)
Fractionation schedules	
10 Gy × 5	10 (19%)
9 Gy × 3	10 (19%)
10 Gy × 3	5 (10%)
7 Gy × 5	5 (10%)
12.5 Gy × 4	3 (6%)
Other (e.g., 7–8 Gy × 3, 12 Gy × 2)	19 (37%)
PTV metrics	
PTV volume (cc), median (IQR)	27 (13–64)
V100 * (%), median (IQR)	95 (95–98)
Maximum dose (%), median (IQR)	118 (112–127)

Abbreviations: OP, oligoprogression; MDRT, metastasis-directed radiation therapy; Gy, gray; PTV, planning target volume. * V100 is the relative volume of the PTV receiving 100% of the prescription dose.

**Table 3:** Univariate logistic regression analysis for maintenance of systemic therapy for at least 6 months and Cox proportional hazards regression for time to next line of systemic therapy.

	Maintenance of Systemic Therapy for ≥ 6 Months			Time to Next Line Systemic Therapy		
	Odds Ratio	95% CI	*p*-Value	Hazard Ratio	95% CI	*p*-Value
Age at time of RT	1.01	0.97–1.06	0.60	0.98	0.95–1.01	0.15
OM at time of RT	2.35	0.71–7.79	0.16	0.82	0.40–1.69	0.60
De novo metastatic at diagnosis	1.78	0.46–6.91	0.41	0.60	0.26–1.39	0.24
No. of treatment lines 1–2 vs. >2	1.50	0.38–5.93	0.56	1.33	0.62–2.83	0.46
Subtype						
HR+/HER2- (Ref)						
HER2+	0.68	0.15–3.03	0.62	1.09	0.44–2.68	0.86
TNBC	0.18	0.02–1.95	0.16	1.22	0.36–4.12	0.75
Bone (Y vs. N)	0.84	0.26–2.72	0.77	1.40	0.69–2.85	0.35
No. of OP lesions treated (1 v >1)	0.47	0.12–1.85	0.28	1.03	0.46–2.31	0.95
BED3	1.01	1.00–10.2	0.30	1.00	0.99–1.00	0.06
BED10	1.01	0.99–1.03	0.46	0.99	0.98–1.00	0.13

Abbreviations: RT, radiation therapy; HR, hormone receptor; HER2, human epidermal growth factor receptor 2; TNBC, triple-negative breast cancer; OP, oligoprogressive; BED, biological effective dose.

**Table 4:** Cox proportional hazards regression for local control, progression-free survival, and overall survival.

	LC			PFS			OS		
	Hazard Ratio	95% CI	*p*-Value	Hazard Ratio	95% CI	*p*-Value	Hazard Ratio	95% CI	*p*-Value
Age at time of RT	0.97	0.87–1.08	0.58	0.98	0.96–1.01	0.25	0.96	0.91–1.02	0.17
OM at time of RT	2.08	0.21–20.27	0.53	0.63	0.32–1.23	0.17	0.75	0.20–2.80	0.66
De novo metastatic at diagnosis	--- *	--- *	--- *	0.75	0.34–1.64	0.46	0.83	0.17–4.03	0.82
No. of treatment lines 1–2 vs. >2	4.78	0.43–52.91	0.20	0.85	0.41–1.78	0.67	2.52	0.67–9.42	0.17
Subtype									
HR+/HER2- (Ref)									
HER2+	2.24	0.30–16.82	0.43	1.20	0.52–2.76	0.67	0.50	0.06–4.02	0.52
TNBC	---	---	---	0.89	0.27–3.00	0.85	---	---	0.99
Bone (Y vs. N)	1.42	0.13–15.66	0.78	1.31	0.67–2.57	0.42	0.56	0.15–2.10	0.39
No. of OP lesions treated (1 v >1)	2.15	0.19–23.94	0.54	1.11	0.52–2.37	0.78	1.61	0.40–6.48	0.50
BED3	0.99	0.99–1.00	0.02	0.99	0.99–1.00	0.02	1.00	0.99–1.01	0.61
BED10	0.80	0.63–1.01	0.06	0.99	0.97–1.00	0.04	1.00	0.97–1.02	0.71

Abbreviations: LC, local control; PFS, progression-free survival; OS, overall survival; RT, radiation therapy; HR, hormone receptor; HER2, human epidermal growth factor receptor 2; TNBC, triple-negative breast cancer; OP, oligoprogressive; BED, biological effective dose. * No local recurrence events were observed in patients with de novo metastatic disease.

## Data Availability

The data that support the findings of this study are available from the corresponding author upon reasonable request.

## Abbreviations

The following abbreviations are used in this manuscript:

OP	Oligoprogressive
MDRT	Metastasis-directed radiation therapy
MBC	Metastatic breast cancer
RT	Radiation therapy
OM	Oligometastases
HR	Hormone receptor
HER2	Human epidermal growth factor receptor 2
TNBC	Triple-negative breast cancer
OP	Oligoprogressive
BED	Biological effective dose
PFS	Progression-free survival
OS	Overall survival
